# Occurrence and characterization of NDM-5-producing *Escherichia coli* from retail eggs

**DOI:** 10.3389/fmicb.2023.1281838

**Published:** 2023-11-23

**Authors:** Yi-Yun Liu, Tong Li, Huiying Yue, Chao Yue, Litao Lu, Junqiang Chen, Haotian Deng, Xun Gao, Jian-Hua Liu

**Affiliations:** ^1^State Key Laboratory for Animal Disease Control and Prevention, Guangdong Laboratory for Lingnan Modern Agriculture, College of Veterinary Medicine, South China Agricultural University, Guangzhou, China; ^2^Key Laboratory of Zoonosis of Ministry of Agricultural and Rural Affairs, Guangdong Provincial Key Laboratory of Veterinary Pharmaceutics Development and Safety Evaluation, Guangzhou, Guangdong, China

**Keywords:** resistance, food, carbapenemase, egg, plasmid

## Abstract

The New Delhi Metallo-β-lactamase (NDM) producing Enterobacterales has been detected from diverse sources but has rarely been reported in retail eggs. In this study, 144 eggshell and 96 egg content samples were collected in 2022 from Guangdong province and were screened for NDM-producing strains. Four *Escherichia coli* strains (ST3014, ST10, ST1485, and ST14747) recovered from two (1.39%, 2 of 144) eggshells and two (2.08%, 2 of 96) egg content samples were identified as *bla*_NDM−5_-positive strains. Oxford Nanopore MinION sequencing and conjugation assays revealed that the *bla*_NDM−5_ gene was carried by IncX3 (*n* = 1), IncI1 (*n* = 1), and IncHI2 (*n* = 2). The IncI1-plasmid-carrying *bla*_NDM−5_ displayed high homology with one plasmid pEC6563-NDM5 from the human clinic, while the IncHI2 plasmid harboring *bla*_NDM−5_ shared highly similar structures with plasmids of animal origin. To the best of our knowledge, this is the first report on the identification of *bla*_NDM−5_-positive bacteria in retail eggs. NDM-producing *E. coli* could be transmitted to humans by the consumption of eggs or direct contact, which could pose a potential threat to human health.

## 1 Introduction

Carbapenemase-resistant Enterobacterales (CRE) have increased rapidly over the last decades and have become an urgent public health threat (El-Gamal et al., 2017). Carbapenem resistance in Enterobacteriaceae is attributed to three dominant carbapenemase enzymes, including New Delhi metallo-beta-lactamases (NDM), *Klebsiella pneumoniae* carbapenemases (KPC), and carbapenem-hydrolyzing oxacillinase-48-type β-lactamases (OXA-48) (Iovleva and Doi, 2017). Among these CRE, NDM-producing strains are highly prevalent around the world, especially in China and South Asia (Wu et al., 2019). At present, 47 variants of NDM have been identified (https://www.ncbi.nlm.nih.gov/pathogens/refgene/#NDM); of these, NDM-1 and NDM-5 remain the most prevalent carbapenemases (Shen et al., 2022; Saravanan et al., 2023). The NDM-5 possesses higher carbapenemase activity than NDM-1 (Hornsey et al., 2011). Currently, the *bla*_NDM−5_ gene has been disseminated to various bacterial species (e.g., *Escherichia coli, K. pneumoniae*, and *Klebsiella aerogenes*), with *E. coli* as the main bacterial host (Nordmann and Poirel, 2019; Jean et al., 2022; Ma et al., 2023).

Plasmid-mediated transmission has facilitated the widespread distribution of the *bla*_NDM−5_ gene among bacteria from various environmental sources and geographical regions. The *bla*_NDM−5_ gene has been found in an array of plasmid replicon types, such as IncX3, IncFII, IncF, IncN, and IncHI2 (Nordmann and Poirel, 2019; Jean et al., 2022; Lv et al., 2022). The IncX3 has long been recognized as the primary carrier for transmission of the *bla*_NDM−5_ gene (Shen et al., 2022); however, recently, there is an increase in IncHI2 plasmid as a carrier of *bla*_NDM−5_ in China (Ma et al., 2021; Zhao Q. et al., 2021; Lv et al., 2022; Wang et al., 2022; He et al., 2023). Alarmingly, IncHI2 has also been found to carry multiple antibiotic resistance genes, including colistin resistance gene (*mcr*), extended-spectrum beta-lactamase, and quinolone resistance genes (Webb et al., 2016; Mmatli et al., 2022).

Although carbapenems have not been approved for food animals, CRE has been continuously detected in pigs, poultry, and animal-derived foods, especially from chickens and poultry products. Eggs are important poultry products that play an essential role in the daily healthy diet of human beings and are the most consumed food all over the world [https://www.who.int/zh/news-room/fact-sheets/detail/salmonella-(non-typhoidal)]. Poultry eggs are also considered as reservoirs and transmission vectors of resistance genes, such as *mcr-1, fosA, qnrS1*, *bla*_CTX − M−1_, *bla*_IMP_, and *bla*_OXA − 48−*like*_ (Benameur et al., 2018; Kapena et al., 2020; Zhang et al., 2021, 2022b; Kanaan et al., 2022; Li et al., 2022). Resistant bacteria and genes in eggs have the risk of spreading to humans through various ways, such as hand-to-egg contact and storing unwashed eggs in fridge. However, the occurrence of clinically important resistant bacteria, NDM-producing Enterobacterales, in eggs has rarely been studied. Hence, we investigated the prevalence of NDM-producing Enterobacterales among egg samples recovered from markets in Guangzhou and characterized the molecular traits of *bla*_NDM_-positive isolates.

## 2 Methods

### 2.1 Sampling

From June to September 2022, 144 non-repetitive egg samples were randomly collected from 29 farmer markets located in four districts (Tianhe, Baiyun, Yuexiu, and Haizhu) of Guangzhou. To ensure diversity in the sampling and prevent repeated sampling from a singular supplier, we selected different stalls within each market, with a maximum of three eggs procured from any single stall. Each sample was placed in a separate sterile sample bag, and all samples were transported to the laboratory in a cool box within 8 h.

### 2.2 Bacterial isolation and detection of carbapenemase-encoding genes

For the isolation of bacteria from eggshells, the surface of eggs was wiped with a sterile swab, and then, the swab was placed into 4 ml sterilized Luria–Bertani (LB) broth medium for enrichment cultivation at 37°C overnight. For the isolation of bacteria from egg content, the eggshell was wiped with gauze with 70% ethanol, followed by being homogenized. During the processing of the first batch, 48 cracked eggs were collided and discarded to avoid cross-contamination (detailed information about all the samples is shown in Supplementary Table S1), and then, the remaining eggs were opened to extract the whole egg content. In total, 1 ml of egg content was dispensed into 4 ml sterilized Luria–Bertani (LB) broth medium and enriched at 37°C overnight with shaking. The overnight cultures of each sample were incubated on MacConkey agar plates supplemented with 0.5 mg/L meropenem, and the plates were incubated at 37°C for 16 h. One to three colonies with different morphologies in each plate were selected for the detection of carbapenemase-encoding genes, *bla*_NDM_, using PCR and DNA sequencing (primers are shown in Supplementary Table S2). All *bla*_NDM_-positive isolates were collected for species identification by direct smear and matrix-assisted laser desorption/ionization–time-of-flight mass spectrometry (MALDI-TOF MS; Bruker Daltonik GmbH, Bremen, Germany).

### 2.3 Antimicrobial susceptibility testing

According to the recommendations of the Clinical and Laboratory Standards Institute, the minimal inhibitory concentrations (MICs) of 19 antimicrobials against NDM-positive isolates were determined using the agar dilution method or broth microdilution (colistin and tigecycline) method. *E. coli* ATCC 25922 was used as a quality control strain. The results of MICs were interpreted according to CLSI (M100-S30) criteria and EUCAST (http://www.eucast.org/clinical_breakpoints/).

### 2.4 Plasmid transferability and stability

Conjugation experiments were performed by broth mating using *bla*_NDM_-positive strains as the donor and a sodium azide-resistant (MIC > 2,000 μg/ml) *E. coli* J53 strain as the recipient. In detail, the donor and recipient strains were incubated separately in LB broth for 4 h, followed by mating the bacterial cultures with a ratio of 1:1 and incubating at 37°C without shaking overnight. A 50-μL overnight mixture was plated onto MacConkey agar plates containing 0.5 mg/L meropenem and 150 mg/L sodium azide, and incubated for 18 h to count and select transconjugants. Conjugation frequency was calculated as the number of transconjugants per recipient. Chemical transformation experiments were performed in those cases that *bla*_NDM_-positive strains failed to conjugate. All transconjugants and transformants were confirmed by PCR (primers are shown in Supplementary Table S2) and antimicrobial susceptibility testing.

The stability of *bla*_NDM−5_-carrying plasmids in host bacteria was performed by a passage in the absence of antibiotic Luria broth (LB). Three single clones of each *bla*_NDM−5_-positive strain were grown in 3 ml LB without antibiotic treatment overnight at 37°C. The overnight culture was daily diluted 1:100 in fresh LB broth for 15 days. Cultures were collected at the end of each of 3 days for streaking on antibiotic-free MacConkey agar plates. Then, 100 colonies were selected, and the presence of *bla*_NDM−5_ and the corresponding plasmids was verified by PCR amplification of *bla*_NDM−5_ and *repA* (primers are shown in Supplementary Table S2). Plasmid retention was calculated as the ratio of strains with *bla*_NDM−5_ and *repA* and over 100 colonies.

### 2.5 Whole-genome sequencing and bioinformatics analysis

Genomic DNAs of four NDM-positive isolates were extracted by using HiPure Bacterial DNA Kit (Magen, Beijing, China), according to the manufacturer's instructions. Whole genomic DNA was sequenced using the Illumina NovaSeq 6000 and MinION platform (Nanopore, UK). Hybrid assembly of complete genomes was carried out using the Unicycler version 0.4.8 (Wick et al., 2017). MLST v2.19 (https://github.com/tseemann/mlst) was applied to the verified sequence type (ST). Center for Genomic Epidemiology (CGE) (http://genomicepidemiology.org/services/) and PubMLST were used to identify antimicrobial resistance genes (ARGs) and plasmid replication types. Prokka software was used to annotate the draft genome (Seemann, 2014). EasyFig tool (http://mjsull.github.io/Easyfig/) was used to draw the genetic context of *bla*_NDM_ and plasmid alignments and comparisons (Sullivan et al., 2011).

### 2.6 Accession numbers

The complete sequences of NDM-positive isolates have been deposited in the GenBank database under accession numbers PRJNA983957.

## 3 Results

### 3.1 Prevalence of NDM-producing enterobacterales in egg samples

From the 144 eggs, 144 eggshell and 96 egg content samples were collected, and 4 *bla*_NDM_-positive isolates were recovered from 2 (1.39%, 2/144) eggshell samples (GD22SC3180PM and GD22SC3312PM) and 2 (2.08%, 2/96) egg content samples (GD22SC3148PM and GD22SC4181PM). These strains were further identified as *E. coli* by MALDI-TOF MS. All the *bla*_NDM_ genes were identified as *bla*_NDM−5_.

### 3.2 Antimicrobial resistance patterns of *bla*_**NDM-5**_-positive *E. coli* strains

Antimicrobial susceptibility testing showed that all four isolates were resistant to most of the antimicrobials, including β-lactams (ampicillin, cefoxitin, cefotaxime, ceftazidime, cefquinome, and imipenem), aminoglycosides (apramycin, neomycin, and streptomycin), fosfomycin, florfenicol, tetracycline, and sulfamethoxazole. However, all isolates remained susceptible to amikacin, colistin, and tigecycline (Supplementary Table S3).

### 3.3 Genotyping and genetic background of *bla*_**NDM-5**_-positive *E. coli* strains

The four *bla*_NDM−5_-positive *E. coli* strains were subjected to short- and long-read sequencing to acquire complete genomes. Sequence analysis revealed that GD22SC3180PM, GD22SC3312PM, GD22SC3148PM, and GD22SC4181PM belonged to ST14747, ST10, ST3014, and ST1485, respectively (Table 1). All *bla*_NDM_-positive isolates harbored multiple antimicrobial resistance genes (ARGs) and plasmid replicon types (Table 1). Aminoglycoside resistance gene, β-lactam resistance gene, florfenicol resistance gene *floR*, macrolide resistance gene *mdf(A)*, and sulfonamide resistance gene *dfrA* were detected in all the *bla*_NDM−5_*-positive E. coli strains*. In addition, the fluoroquinolone resistance gene *qnrS* and rifampicin resistance gene *arr-3* were identified in two isolates (GD22SC3312PM and GD22SC4181PM). GD22SC3180PM additionally harbored tetracycline resistance gene *tet*(A), fosfomycin resistance gene *fosA3*, and lincomycin resistance gene *lnu(F)*.

**Table 1:** Characteristics of *bla*_NDM−5_-positive isolates.

**Isolates**	**Source**	**Organism**	**Sequence type**	**Resistance genes**	**Plasmid types**	**Plasmid size (bp)**	**Imipenem MIC (μg/mL)**	**Conjugation frequency^b^**
GD22SC3148PM	Egg content FM16^a^	*E. coli*	ST3014	*aadA2*, **bla_NDM−5_**, *cmlA1, dfrA12, floR, mdf(A), sitABCD, sul2, sul3*	IncFIA, IncFIB(AP001918), IncFIC(FII), **IncX3**	–	4	N.D.
GD22SC3180PM	Egg shells FM17	*E. coli*	ST14747	*aac(3)-IId, aac(3)-IVa, aadA17, aadA5, aph(3^′′^)-Ib, aph(3′)-Ia, aph(6)-Id*, *bla*_CTX − M−55_, **bla**_NDM−5_, *dfrA17, floR, fosA3, lnu(F), mdf(A), mph(A), sitABCD, sul1, sul2, sul3, tet*(A)	IncFIB(AP001918), IncFII(pHN7A8), **IncI1**, p0111	107,617	8	(3.75 ± 1.33) × 10^−6^
GD22SC3312PM	Egg shells FM20	*E. coli*	ST10	***arr-3***, ***aac(3)-IV***, ***aph(3**^**′′**^**)-Ib**, aph(3′)-Ia, aph(4)-Ia*, ***aph(6)-Id***, **bla**_CTX − M−55_, **bla**_NDM−5_, **bla**_OXA − 10_, *aadA2, dfrA12, **dfrA14***, ***floR***, *lnu(F), **mdf(A)***, ***qnrS1***, ***sitABCD***, ***sul1***, ***sul3***	Col156, IncB/O/K/Z, IncFIB(AP001918), IncFIC(FII), **IncHI2**, IncHI2A, p0111	263,640	4	(1.76 ± 0.16) × 10^−5^
GD22SC4181PM	Egg content FM25	*E. coli*	ST1485	***arr**-3, aac(3)-IVa, aph(3′)-Ia, aph(4)-Ia*, *bla*_NDM−5_, *bla*_OXA − 10_, *bla*_TEM − 1B_, *cmlA1, dfrA14, floR, mdf(A), qnrS1, sitABCD*	IncFIA, IncFIB(AP001918), IncFIC(FII), IncFII, IncHI2A, **IncHI2**, p0111	–	4	N.D.

Bold, *bla*_NDM−5_-positive plasmids and their resistance genes.

a FM, Famers' market.

b Average ± Standard error (SE).

N.D. means that conjugation frequency cannot be measured.

### 3.4 Characterization of *bla*_**NDM-5**_-carrying plasmids

**Figure 1:**
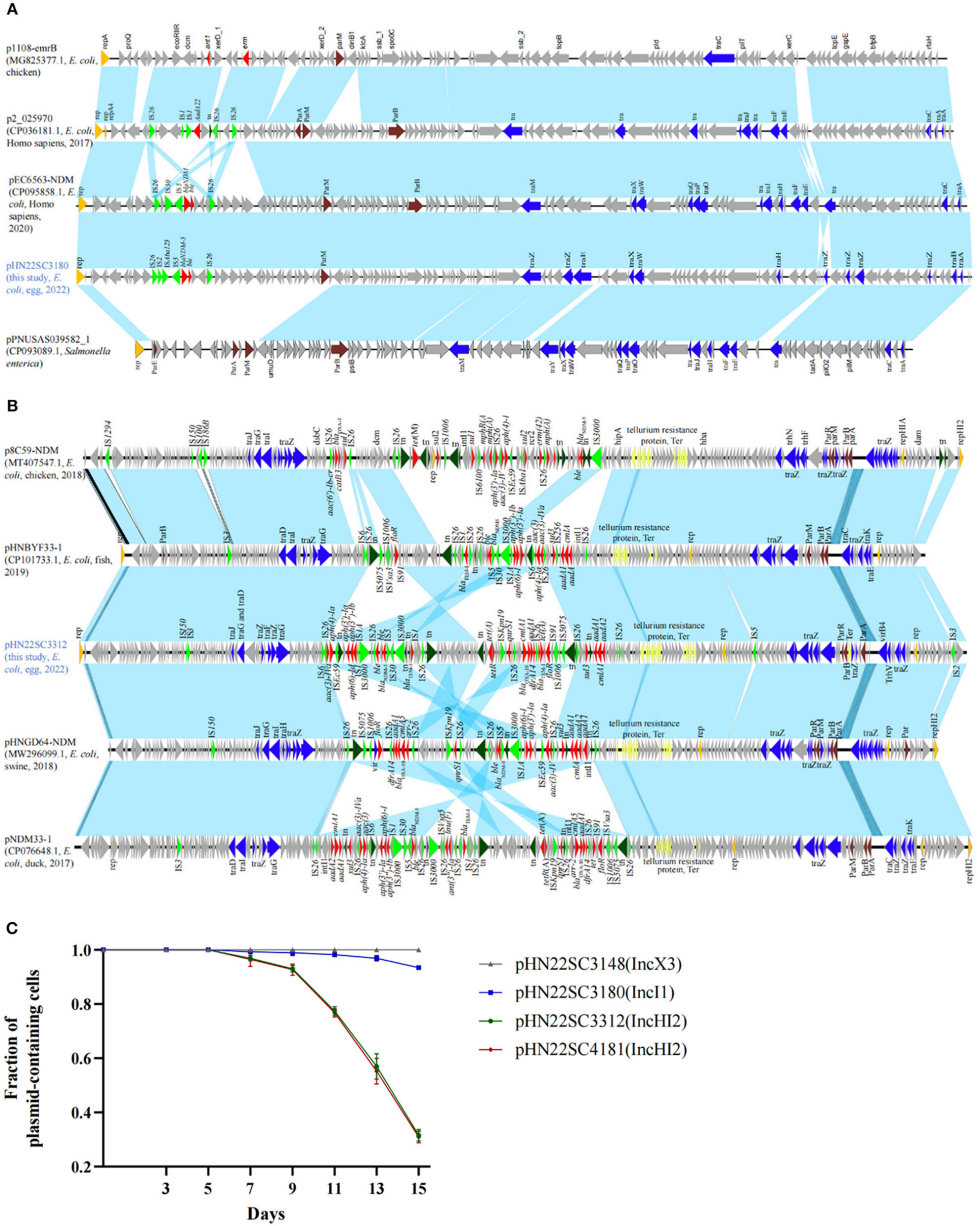
Comparison and stability of *bla*_NDM−5_-carrying plasmids. **(A)** *bla*_NDM−5_-harboring IncI1 plasmids in this study with other similar plasmids. p1108-emrB (NZ_MG825377.1), p2_025970 (CP036181.1), pPNUSAS039582_1 (CP093089.1), and pEC6563-NDM5 (CP095858.1). **(B)** *bla*_NDM−5_-harboring IncHI2 plasmids in this study with other similar plasmids. p8C59-NDM (MT407547.1), pHNBYF33-1 (CP101733.1), pHNGD64-NDM (MW296099.1), and pNDM33-1 (CP076648.1). **(C)** Stability of *bla*_NDM−5_-carrying plasmids in their corresponding host bacteria. Error bars represent standard deviations (*n* = 3).

Bioinformatics analysis revealed that there were three types of replicons carrying the *bla*_NDM−5_ gene, namely, IncX3 (pHN22SC3148), IncI1 (pHN22SC3180), and IncHI2 (pHN22SC3312 and pHN22SC4181) (Table 1). The complete sequence of IncI1 plasmid pHN22SC3180 is a 107,617 circular molecule with a GC content of 47% (Figure 1A). The backbone regions of the pHN22SC3180 displayed the highest similarity to plasmid pEC6563-NDM5 (CP095858.1, urine, Homo sapiens, *E. coli*, Zhejiang, China), with 100% identity and 99.0% coverage (Figure 1A) (Zhang et al., 2022a), but had low similarity to the other four *bla*_NDM−5_-bearing IncI1 plasmids deposited in the NCBI database (Supplementary Table S4). The plasmid pHN22SC3312 (IncHI2) was 263,640 bp in size with a GC content of 54% (Figure 1B) and had a high degree of homology with pHNGD64-NDM (MW296099.1, pig, *E. coli*, Guangdong, China) with 100% identity and 98.0% coverage, pNDM33-1 (CP076648.1, duck, *E. coli*, Guangdong, China) with 99.99% identity and 99.0% coverage, and pHNBYF33-1 (CP101733.1, fish, *E. coli*, Guangdong, China) with 99.99% identity and 98.0% coverage (Figure 1B). Furthermore, in addition to *bla*_NDM−5_, the variable region of pHN22SC3312 contained several antibiotic resistance genes [e.g., *bla*_OXA − 10_, tet*(A), qnrS1, floR, sul3, aadA2*, and *aph(4)-Ia*].

### 3.5 The biological features of *bla*_**NDM-5**_-carrying plasmids

To evaluate the transferability of the *bla*_NDM−5_ gene, all four *bla*_NDM−5_ positive strains were conducted on a conjugation assay. The *bla*_NDM−5_-carrying plasmids were successfully transferred to recipients *E. coli* J53 at a frequency of 10^−5^-10^−6^. The imipenem MICs of the transconjugants were 2–4 μg/ml, which were 32–64-fold the MICs of the recipient (Supplementary Table S3). To evaluate the stability of *bla*_NDM−5_-carrying plasmids, we performed passage with the four *bla*_NDM−5_-positive strains in antibiotic-free Luria broth. The stability of the IncX3 plasmid pHN22SC3148 was 100% in the absence of antibiotic after 15 days (i.e., ~150 generations) in the natural host GD22SC3148PM, while IncI1 plasmid pHN22SC3180 and IncHI2 plasmids, pHN22SC3312 and pHN22SC4181, were gradually lost from their corresponding host strains after 7 days of passage, with 93.40, 31.60, and 30.90% retention after 15 days, respectively (Figure 1C). Thus, in the absence of antibiotic selection, IncX3 plasmid pHN22SC3148 is stable in the original isolate, and the other three plasmids (IncHI2 and IncI1) are less stable in the host strains.

### 3.6 Genetic environments of *bla*_**NDM-5**_ genes in IncI1 and IncHI2

**Figure 2:**
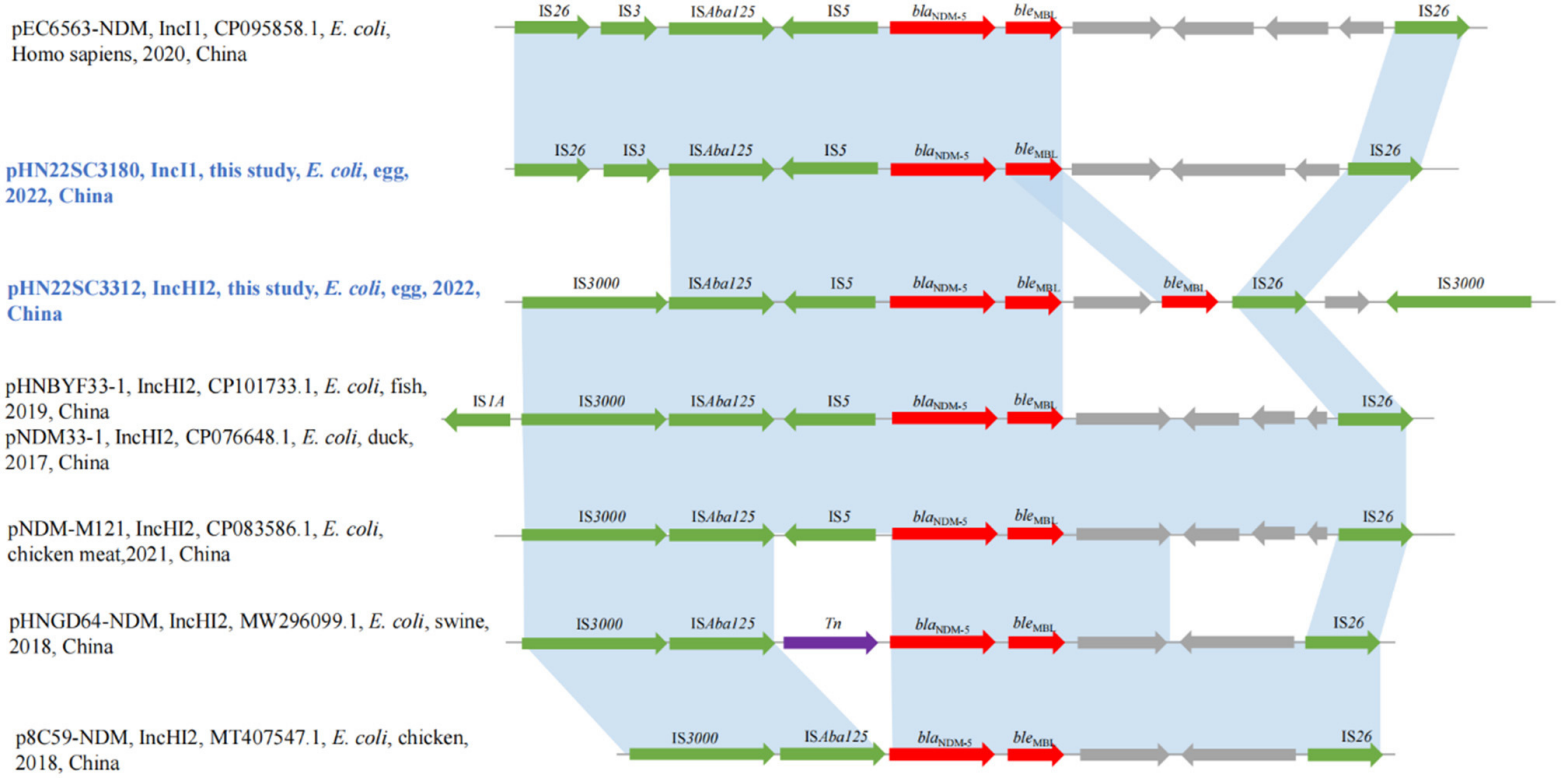
The genetic environment of *bla*_NDM−5_ genes.

The genetic context of the *bla*_NDM−5_ gene in IncI1 plasmid pHN22SC3180 was IS*26*-IS3-IS*Aba125*-IS*5*-*bla*_NDM−5_-*ble*_MBL_-trpF-*dsbD*-IS*26*, which was similar to the genetic environment recently discovered in IncI1 plasmid pEC6563-NDM5-carrying *bla*_NDM−5_ (GenBank accession no. CP095858.1) (Figure 2). Although the *bla*_NDM−5_ region (IS*26*-IS*3*-IS*Aba125*-IS*5*-*bla*_NDM−5_-*ble*_MBL_-trpF-*dsbD*-IS*26*) was surrounded by two copies of IS*26* with the same direction, no circular intermediate was obtained in this study, similar to previous report (Zhang et al., 2022a). Moreover, the genetic contexts of *bla*_NDM−5_ in IncHI2 plasmid pHN22SC3312, IS*3000*-IS*Aba125*-IS*5*-*bla*_NDM−5_-*ble*_MBL_-trpF-*ble*_MBL_-IS*26*-*dsbD*-IS*3000*, were highly similar to other *bla*_NDM−5_-harboring IncHI2 plasmids, pNDM-M121 (GenBank accession no. CP083586.1), pHNBYF33-1 (GenBank accession no. CP101733.1), and pNDM33-1 (GenBank accession no. CP076648.1), except for the presence of two copies of *ble*_MBL_ downstream of *bla*_NDM−5_ in pHN22SC3312 in this study (the information of all egg samples is shown in Supplementary Table S1).

## 4 Discussion

To date, *bla*_NDM_-positive Enterobacterales have been identified in various sources, including food animals, pets, human beings, animal foods, vegetables, and the environment (Zhai et al., 2020; Huang et al., 2023; Ma et al., 2023). However, there are few reports of NDM-producing bacteria in egg sources, except for one study, which reported the presence of *bla*_NDM_-positive *Salmonella enterica* in eggs from Iraq (Kanaan et al., 2022). To the best of our knowledge, this is the first report of NDM-5-producing Enterobacterales in retail egg samples from China. As eggs are an important food in the human diet and its consumption continues to increase, the NDM-positive Enterobacterales in eggs have the risk of spreading to humans via the food chain and even hand–egg contact.

Previous studies revealed that IncX3 is the most epidemiologically successful vehicle for spreading *bla*_NDM_-5 (Zhang et al., 2019; Zhao Q. Y. et al., 2021; Ma et al., 2023). *bla*_NDM−5_-bearing IncX3 plasmids are widely distributed in animals, human beings, and environments worldwide (Lv et al., 2022; Ma et al., 2023). The IncX3 plasmids carrying *bla*_NDM−5_ in this study further confirmed the importance of the IncX3 plasmid by acting as a vehicle for *bla*_NDM−5_ transfer. The *bla*_NDM−5_-positive IncX3 plasmids can be stably inherited in the original isolate (Figure 1C), which may partly explain the rapid global dissemination of *bla*_NDM−5_-bearing IncX3 plasmids (Ma et al., 2020).

In this study, we also detected the IncI1 plasmid (pHN22SC3180) and IncHI2 plasmid (pHN22SC3312) carrying the *bla*_NDM−5_ gene. IncI1 is an epidemic plasmid and can carry many resistance genes, especially the extended-spectrum beta-lactamase gene *bla*_CTX_, which has widely spread in patients and animals (Yang et al., 2014; Chong et al., 2018; Carattoli et al., 2021; Liu et al., 2021). However, the reports of the *bla*_NDM−5_-bearing IncI1 plasmid are few, and *bla*_NDM−5_-bearing IncI1 plasmid has just been detected in isolates from clinical and duck samples in China (Zhao Q. Y. et al., 2021; Dong et al., 2022; Zhang et al., 2022a). Of note, by searching through the NCBI database, we found only five *bla*_NDM−5_-bearing IncI1 plasmids, four of which were clinical samples isolated from China in recent years, implying that the prevalence and risk of *bla*_NDM−5_-bearing IncI1 in the clinic might be underestimated and need further investigation.

IncHI2 is a wide host plasmid and acts as an important vector for the dissemination of multiple ARGs, especially *mcr-1* (Webb et al., 2016; Liu and Liu, 2018; Wu et al., 2018; Cao et al., 2020). To date, IncHI2-type plasmids carrying *bla*_NDM−5_ have only been detected in strains recovered from chicken, duck, pig feces, and freshwater fish (Ma et al., 2021; Zhao Q. Y. et al., 2021; Lv et al., 2022). These IncHI2-*bla*_NDM−5_ plasmids mainly spread regionally in Guangdong province in China but have also spread to other regions (Wang et al., 2022; He et al., 2023). The high similarity of the *bla*_NDM−5_-bearing IncHI2 plasmids in eggs and other origins suggested that these plasmids are spreading. However, IncHI2-type plasmids carrying *bla*_NDM−5_ are not stable in bacterial hosts (Figure 1C). The increasing occurrence of IncHI2-type plasmids carrying *bla*_NDM−5_ in China might be associated with the co-selection by other antimicrobials as IncHI2 plasmids usually carry various antimicrobial resistance genes.

While our findings indicate a slightly higher prevalence of *bla*_NDM_-positive isolates in egg contents (2.08%, 2/96) compared with eggshells (1.39%, 2/144), this study is not without limitations. The exclusion of 48 egg content samples may influence the overall contamination rates. Furthermore, the sample size, hovering around a hundred, does not offer a comprehensive representation of the prevalence of *bla*_NDM_ in egg samples, highlighting the need for continuous surveillance.

## 5 Conclusion

In summary, to the best of our knowledge, we report the first case of Enterobacterales carrying *bla*_NDM−5_ of retail eggs in China. The *bla*_NDM−5_-bearing plasmids displayed high homology with those of plasmids from other sources. Of note, the IncHI2 plasmids carrying both carbapenem and multiple resistance genes showed an increasing trend that pose another threat to human health. Considering the clinical importance of carbapenem together with the fact that the consumption of eggs is substantial in our diet, the carbapenem resistance in eggs has the risk to spread to humans through the food chain or contact with the contaminant. Continued monitoring of carbapenem resistance in eggs is urgently needed.

## Data availability statement

The datasets presented in this study can be found in online repositories. The names of the repository/repositories and accession number(s) can be found in the article/Supplementary material.

## Author contributions

Y-YL: Investigation, Writing—original draft, Writing—review & editing. TL: Formal analysis, Investigation, Writing—original draft. HY: Investigation, Writing—original draft. CY: Investigation, Writing—original draft. LL: Formal analysis, Writing—original draft. JC: Formal analysis, Writing—original draft. HD: Formal analysis, Writing—original draft. XG: Formal analysis, Writing—original draft. J-HL: Conceptualization, Funding acquisition, Writing—review & editing.
